# Oleanolic Acid, a Compound Present in Grapes and Olives, Protects against Genotoxicity in Human Mammary Epithelial Cells

**DOI:** 10.3390/molecules200813670

**Published:** 2015-07-28

**Authors:** Cristina Sánchez-Quesada, Alicia López-Biedma, José J. Gaforio

**Affiliations:** 1 Immunology Division, Department of Health Sciences, Faculty of Experimental Sciences, University of Jaén, Campus las Lagunillas s/n, 23071 Jaén, Spain; E-Mails: csquesad@ujaen.es (C.S.-Q.); albiedma@ujaen.es (A.L.-B.); 2 Centro de Estudios Avanzados en Olivar y Aceites de Oliva, University of Jaén, Campus las Lagunillas s/n, 23071 Jaén, Spain; 3 Agrifood Campus of International Excellence, ceiA3, University of Jaén, Campus las Lagunillas s/n, 23071 Jaén, Spain

**Keywords:** virgin olive oil, wine, maslinic acid, MCF7, MDA-MB-231, MCF10A, chemopreventive, antitumoral

## Abstract

Oleanolic acid (AO) and maslinic acid (MA) are constituents of the skins of different fruits, including olives and white or red grapes. Although both compounds are known to have beneficial properties against different types of cancers, thus far, there are no studies about their chemopreventive effects in human breast cancer. Thus, we sought to elucidate whether both compounds possess chemopreventive activity. Two cell lines of human breast cancer cells and one noncancerous human mammary epithelial cells were used to determine the effects of OA and MA. The results showed that OA inhibited the proliferation and increased the oxidative stress of highly invasive cells. Additionally, OA decreased oxidative stress and oxidative damage to the DNA in human mammary epithelial cells. These results suggest that OA could act as a chemopreventive agent in human breast cancer and could inhibit the proliferation of highly invasive breast cancer cells.

## 1. Introduction

The triterpenoids are natural compounds that are widely distributed in the skin and seeds of different edible fruits, such as olives and grapes from *Vitis vinifera*. Oleanolic acid (OA) and maslinic acid (MA) are two of the main triterpenes found in these fruits; in addition, they are also present in both virgin olive oils and wine, especially red wine [1,2,3,4,5,6,7].

The traditional Mediterranean diet, characterized by the consumption of foods such as grapes, wine, must, raisins, olives and virgin olive oil, has been associated with a low incidence of breast cancer [8]. Current knowledge highlights the role of triterpenes in the prevention of certain cancers, including breast cancer [9,10,11,12,13]. Previously, it has been described that oleanolic acid and maslinic acid possess cardioprotective effects [14,15], anti-inflammatory effects [16,17], and antitumor properties in human prostate cancer cells [18], hepatocellular carcinoma cells [19], human pancreatic cells [20], and colon cancer cells, among others [21,22]. However, there are no studies about the potential chemopreventive effects of oleanolic and maslinic acids in human breast cells. We hypothesized that the chemopreventive effects of Mediterranean diet consumption against breast cancer may be due, at least in part, to the biological actions exerted by these compounds. To demonstrate this hypothesis, we have used the following well-characterized human breast cell lines: MCF10A human mammary epithelial cells, highly invasive MDA-MB-231 human breast cancer cells, and finally, minimally invasive MCF7 human breast cancer cells.

## 2. Results

### 2.1. Cytotoxicity Effects

**Figure 1:**
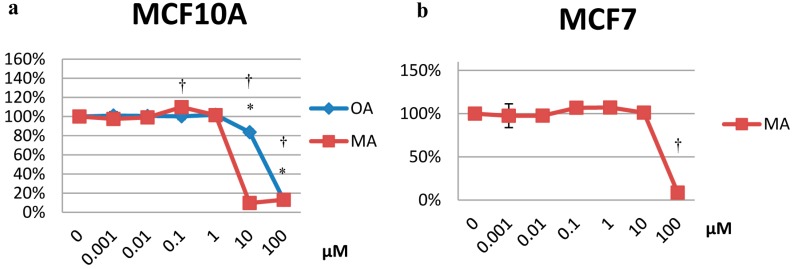

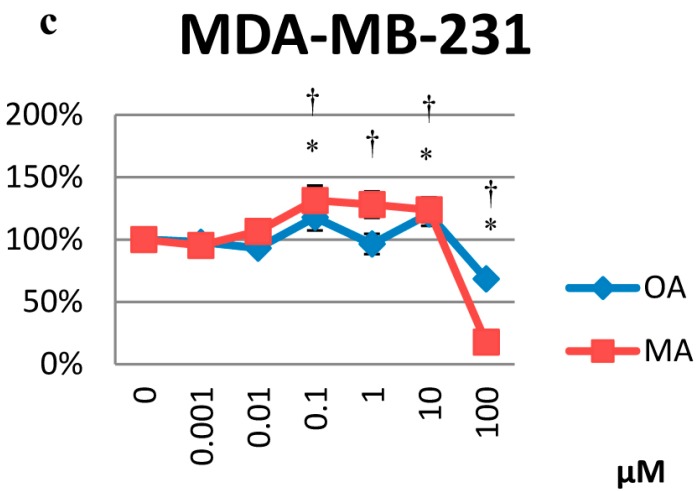
Cytotoxicity of OA and MA from 0.001 µM to 100 µM in MCF10A cells (**a**), MCF7 cells (**b**) and MDA-MB-231 cells (**c**) at 24 h. Values represent the mean ± SEM of three independent experiments. Statistically significant differences are represented by (*) for OA and (†) for MA at *p* < 0.05 compared to the untreated control.

The results are expressed as the percentage of cell survival with respect to the untreated control, which was set as 100%. For MCF10A cells, both OA and MA at 10 and 100 µM promoted cell death (cell survival was 83% and 13% for OA and 9% and 13% for MA, respectively) (Figure 1a). For MCF7 cells, MA induced a strong cytotoxic effect at 100 µM (8% survival) (Figure 1b). MDA-MB-231 cells treated with the two acids showed a marked cytotoxic effect for OA or MA at 100 µM (68% and 17% survival, respectively). MA concentrations between 0.01 µM and 10 µM appeared to promote cell survival (Figure 1c).

In the human mammary epithelial cells, both compounds were cytotoxic at the highest concentrations. However, for MCF7, which is a multi-drug-resistant cancer cell line, only MA was capable of promoting cell death. OA did not significantly promote cytotoxicity in MCF7 cells, according to our previous study [10]. Our results agree with Shan *et al.*, who showed that OA did not strongly inhibit the growth of MCF7 cells [23]. In MDA-MB-231 cells, other studies of different plant extracts (which contain OA) have described antiproliferative effects [24,25]. Ponou *et al.* showed that isolated OA did not promote cytotoxicity at a maximum concentration of 200 µM [26], while we observed cytotoxicity at 100 µM.

### 2.2. Effects on Proliferation 

**Figure 2:**
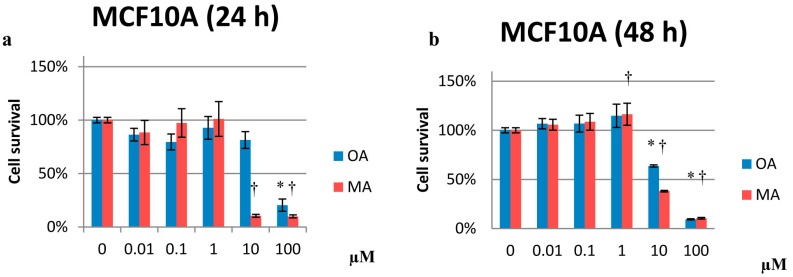

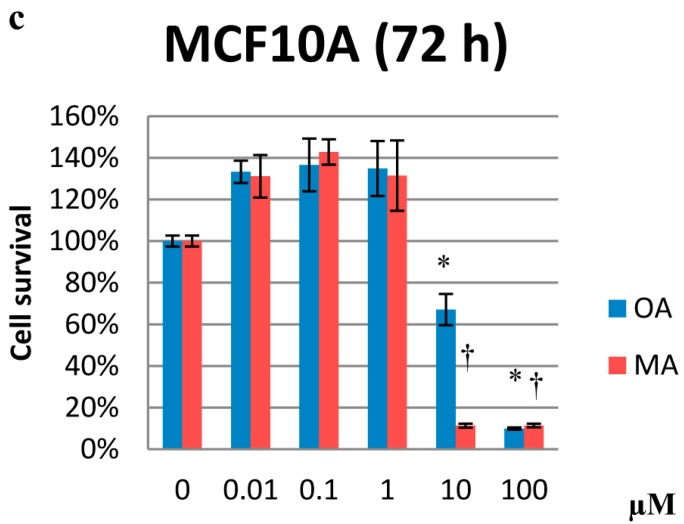
Percentage of cell proliferation in MCF10A cells after treatment with 0.01 µM to 100 µM OA or MA at 24 (**a**); 48 (**b**) and 72 h (**c**). Values represent the mean ± SEM of three independent experiments. Statistically significant differences are represented by (*) for OA and (†) for MA at *p* < 0.05 with respect to the untreated control.

The results are expressed as the percentage of cell survival with respect to the untreated control, which was set as 100%. MA at 10 and 100 µM had antiproliferative effects for MCF10A cells at 24, 48, and 72 h (10%, 38% and 11% cell survival for 10 µM and 9%, 10% and 11% for 100 µM, respectively) (Figure 2).

**Figure 3:**
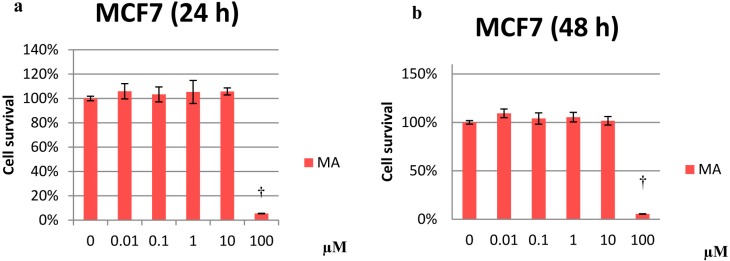

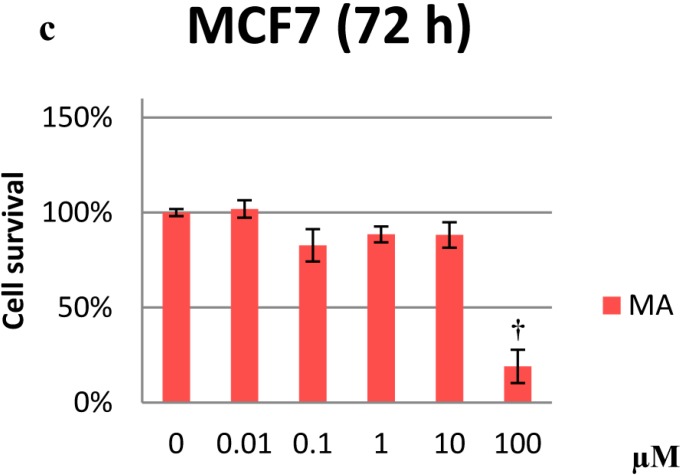
Percentage of cell proliferation in MCF7 cells after treatment with 0.01 µM to 100 µM MA at 24 (**a**); 48 (**b**) and 72 h (**c**). Values represent the mean ± SEM of three independent experiments. Statistically significant differences are represented by (†) for MA at *p* < 0.05 with respect to the untreated control.

**Figure 4:**
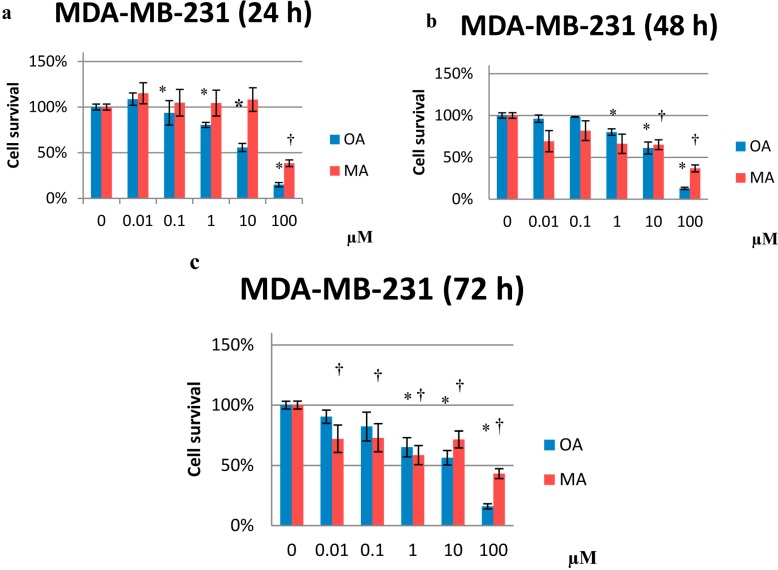
Percentage of cell proliferation in MDA-MB-231 cells after treatment with 0.01 µM to 100 µM OA or MA for 24 (**a**); 48 (**b**) and 72 h (**c**). Values represent the mean ± SEM of three independent experiments. Statistically significant differences are represented by (*) for OA and (†) for MA at *p* < 0.05 with respect to the untreated control.

In MCF10A cells, OA inhibited proliferation at 10 and 100 µM after 48 and 72 h of treatment (~65% and 9% cell survival, respectively, at both time points) (Figure 2b,c). For MCF7 cells, MA was antiproliferative only at 100 µM (Figure 3). In MDA-MB-231 cells, OA inhibited proliferation in a dose-dependent manner at all treatment exposure times (Figure 4). Similarly, OA and MA at 10 and 100 µM inhibited proliferation in human mammary epithelial cells. However, at low concentrations, OA and MA appeared to increase the proliferation of the human mammary epithelial cells over time.

Notably, OA and MA were able to inhibit proliferation in a dose-dependent manner at all of the time exposures assayed in highly invasive breast cancer cells (MDA-MB-231) (Figure 4).

### 2.3. Effects on the Cell Cycle 

The results are expressed as the percentage of cells in the different phases of the cell cycle. For MCF10A cells, OA treatment resulted in an increase in cells in the G0/G1 phase at 10 µM with respect to the control and a decrease in the G2/M phase. MA treatment resulted in a dramatic increase in the sub-G0/G1 phase at 10 µM (65%) with respect to the control (0.4%), and consequently resulted in a decrease in the other phases. At 10 µM, both compounds affected the cell cycle of MCF10A cells (Table 1).

**Table 1:** Distribution of cells in phases of the cell cycle for MDA-MB-231 and MCF10A cells treated with OA and MA at 0.1 µM, 1 µM, and 10 µM at 24 h. Values represent the mean ± SEM of three independent experiments. Statistically significant differences are represented by (*) at *p* < 0.05 with respect to the untreated control.

	Percentage of Cells							
	MDA-MB-231				MCF10A			
	Sub-G0/G1	G0/G1	S	G2/M	Sub-G0/G1	G0/G1	S	G2/M
Control	0.84 ± 0.16	65.38 ± 1.25	16.18 ± 1.08	16.44 ± 2.05	0.39 ± 0.15	58.22 ± 2.93	15.90 ± 1.43	24.54 ± 1.51
OA 0.1 µM	0.75 ± 0.25	59.04 ± 2.99 *	17.31 ± 1.58	21.89 ± 2.09 *	0.54 ± 0.27	56.86 ± 4.60	16.54 ± 2.83	24. 27 ± 1.18
OA 1 µM	0.78 ± 0.25	59.73 ± 1.98	17.60 ± 1.66	20.76 ± 1.54	0.37 ± 0.15	58.23 ± 3.63	15.68 ± 1.86	25.08 ± 1.23
OA 10 µM	0.76 ± 0.37	61.06 ± 1.85	15.04 ± 1.46	21.36 ± 0.91 *	0.84 ± 0. 25	71.45 ± 6.63 *	11.58 ± 2.54	15.14 ± 4.22 *
MA 0.1 µM	1.42 ± 0.49	61.88 ± 0.73	16.86 ± 2.25	19.28 ± 1.61	0.37 ± 0.13	58.77 ± 1.75	15.17 ± 0.20	24.43 ± 2.26
MA 1 µM	0.72 ± 0.49	61.90 ± 0.52	16.66 ± 1.13	19.59 ± 0.84	0.59 ± 0.16	58.81 ± 3.82	16.58 ± 2.65	22.92 ± 1.88
MA 10 µM	0.72 ± 0.01	62.36 ± 0.65	16.56 ± 1.12	19.75 ± 1.64	64.68 ± 1.92 *	21.96 ± 1.82 *	8.01 ± 1.15*	4.56 ± 1.24 *

We have discussed the importance of the different concentrations of treatments used in experiments [27], and our results show that high concentrations of these compounds could promote cell death in human mammary epithelial cells. For MDA-MB-231 cells, OA treatment resulted in a decrease in the number of cells in G0/G1 and an increase in G2/M at 0.1 µM with respect to the control. At 10 µM, OA increased the number of cells in the G2/M phase with respect to the control. MA treatment did not result in a significant difference in MDA-MB-231 (Table 1) or MCF7 cells (data not shown). These results suggest that MA affects the cell cycle of MCF10A cells, increasing the Sub-G0/G1 ratio. This increase could be due to pro-apoptotic effects. To assess this apoptotic effect, our group studied the apoptosis-promoting effects of these compounds in the three breast cell lines.

### 2.4. Analysis of Apoptosis

The percentages of living, apoptotic, and necrotic cells are represented with respect to the total, which was set as 100% (Table 2).

For MCF10A cells, 10 µM OA resulted in a high percentage of apoptotic cells with respect to the control. MA at 10 µM increased the rate of apoptotic cells. For MDA-MB-231 cells, statistically significant differences were not found, but 1 µM OA resulted in a slight increase in the apoptotic cell rate (Table 2). MA treatment in MCF7 cells did not result in a significant difference with respect to the control (data not shown).

**Table 2:** Apoptosis of MDA-MB-231 and MCF10A cells treated with OA or MAS at 0.1 µM, 1 µM and 10 µM at 24 h. Values represent the mean ± SEM of three independent experiments. Statistically significant differences are represented by (*) at *p* < 0.05 with respect to the untreated control.

	Percentage of Cells					
	MDA-MB-231			MCF10A		
	Live	Apoptotic	Death	Live	Apoptotic	Death
Control	87.64 ± 3.16	8.92 ± 2.15	1.33 ± 0.48	92.43 ± 1.43	5.92 ± 1.40	1.63 ± 0.53
OA 0.1 µM	90.66 ± 4.28	8.43 ± 4.04	0.90 ± 0.36	94.43 ± 0.71	3.57 ± 1.31	1.97 ± 0.61
OA 1 µM	86.72 ± 3.27	11.83 ± 3.28	1.43 ± 0.24	94.91 ± 0.74	2.16 ± 0.79	2.90 ± 1.00
OA 10 µM	88.22 ± 2.78	10.20 ± 3.36	1.56 ± 0.61	70.40 ± 16.09	17.18 ± 8.22 *	12.41 ± 7.89
MA 0.1 µM	90.81 ± 3.29	8.12 ± 2.65	1.05 ± 0.67	92.38 ± 2.01	6.35 ± 2.30	1.26 ± 0.33
MA 1 µM	89.43 ± 5.38	7.70 ± 3.06	2.85 ± 2.33	92.35 ± 1.30	5.80 ± 1.84	1.83 ± 0.63
MA 10 µM	88.86 ± 2.41	10.13 ± 2.16	0.98 ± 0.30	5.64 ± 2.31	78.17 ± 8.92 *	16.17 ± 7.01

MA and OA at the highest concentrations caused apoptosis in MCF10A cells, while concentrations lower than 10 µM did not appear to promote apoptosis. However, in both breast cancer cell lines, neither OA nor MA produced a dramatic increase in apoptosis; only 1 µM OA slightly increased the apoptotic ratio in MDA-MB-231 cells. This slight increase could correspond with the proliferation observed, where OA decreased the proliferation in a dose-dependent manner over time.

### 2.5. Effects on the Intracellular ROS Level

**Figure 5:**
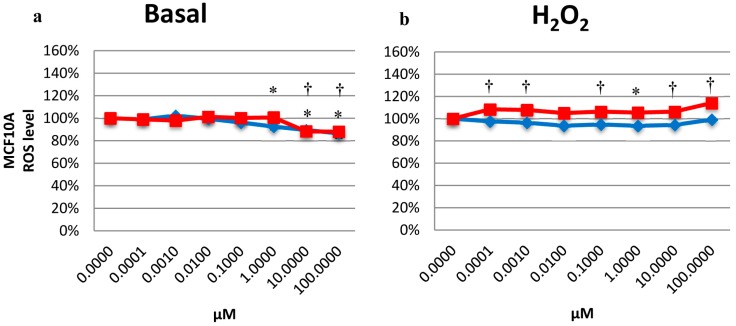

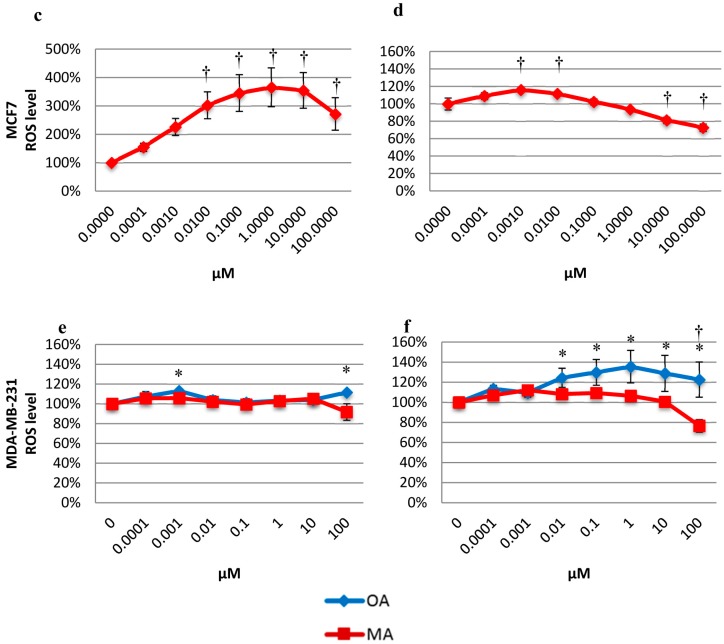
The ROS levels present in MCF10A cells in the basal state (**a**) and with H_2_O_2_ burst (**b**); in MCF7 cells in the basal state (**c**) and with H_2_O_2_ burst (**d**); and in MDA-MB-231 cells in the basal state (**e**) and with H_2_O_2_ burst (**f**) after treatment with OA or MA from 0.0001 µM to 100 µM for 4 h. Values represent the mean ± SEM of three independent experiments. Statistically significant differences are represented by (*) for OA and (†) for MA at *p* < 0.05 with respect to the untreated control.

In MCF10A cells treated with OA and MA, the levels of ROS decreased from 1 µM to 100 µM OA and from 10 to 100 µM for MA (Figure 5a). MA treatment in MCF7 cells increased the ROS levels in a dose-dependent manner (Figure 5c). In MDA-MB-231, OA treatment resulted in an increase in the ROS levels at 0.001 µM and 100 µM, while MA did not alter the ROS levels at any concentration tested (Figure 5d).

To induce intracellular oxidative stress, H_2_O_2_ was added before the fluorescence measurement. Figure 5b shows a decrease in the ROS levels in MCF10A cells for OA; however, this difference was statistically significant only at 1 µM. MA treatment increased the ROS levels in MCF10A cells at almost all concentrations (Figure 5b). For MCF7 cells, MA appeared to increase the ROS levels at lower concentrations (Figure 5d). The ROS levels in MDA-MB-231 cells increased with OA treatment from 0.01 µM to 100 µM, while MA treatment did not result in any statistically significant differences with respect to the control, except for 100 µM, which decreased the ROS level (Figure 5f).

OA had a protective effect on MCF10A cells. It diminished ROS levels in the basal state, and when oxidative stress was induced, OA continued protecting the cells, reducing their sensitivity to oxidative stress. ROS can act as a trigger for carcinogenesis by permanent damage of DNA, causing mutations in p53, the tumour suppressor gene, which is frequently mutated (in up to 50%) [28]. In this way, OA could act like an antioxidant, protecting cells in an oxidative stress microenvironment, which could promote carcinogenesis [27,28]. To assess this theory, our group studied the effects of OA and MAS in H_2_O_2_-induced DNA damage. 

Although MA did not have this effect in MCF10A cells, it resulted in a strong increase in oxidative stress in MCF7 cells in a dose-dependent manner, which continued when oxidative stress was induced. In MDA-MB-231 cells, both compounds exerted this pro-oxidative effect. In the basal state, lower concentrations of OA appeared to increase the oxidative stress in MDA-MB-231 cells. In addition, when intracellular oxidative stress was induced by adding H_2_O_2_, OA dramatically increased the oxidative stress, approximately 30% more than the control. MA had the same effect but to a lesser extent. Therefore, OA had a protective role against oxidative stress in human mammary epithelial cells, while it had a pro-oxidant role in the highly invasive breast cancer cells. This pro-oxidant role in breast cancer cells could be important, considering that high enough levels of ROS may inhibit carcinogenesis by enhancing p53 expression and inducing apoptosis in tumour cells [28]. To corroborate these effects in ROS levels, antioxidant catalase (CAT) enzyme activity was evaluated.

### 2.6. Determination of CAT Activity

**Figure 6:**
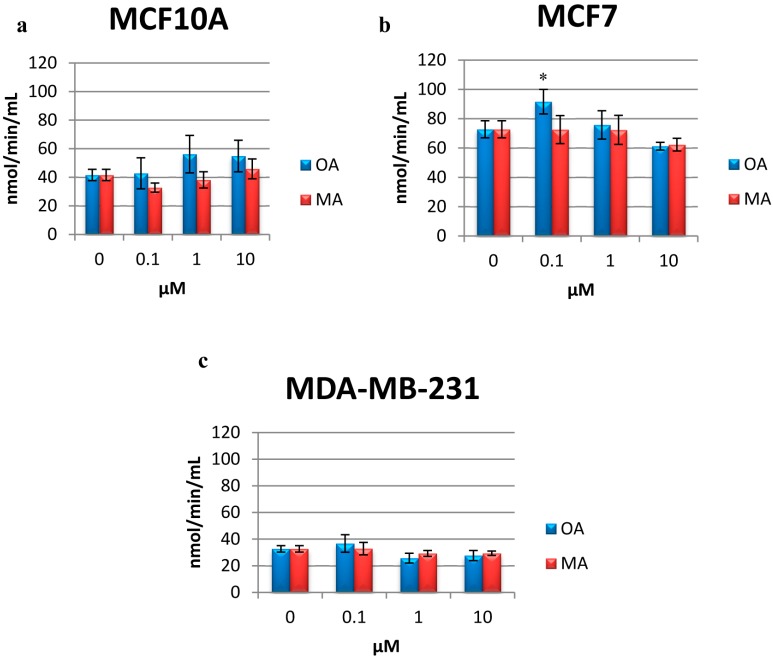
CAT activity in MCF10A cells (**a**); MCF 7 cells (**b**) and MDA-MB-231 cells (**c**) treated with OA or MA at 0.1 µM, 1 µM and 10 µM for 4 h. Values represent the mean ± SEM of three independent experiments. Statistically significant differences are represented by (*) for OA at *p* < 0.05 with respect to the untreated control.

The activity of CAT measured in MCF10A cells after OA and MA treatment showed no statistically significant differences with respect to the control (Figure 6a).

In MCF7 cells, 0.1 µM OA increased CAT production significantly but appeared to decrease its production at higher concentrations. While 0.1 µM of OA was not assayed by Allouche, *et al.* [10], 1 µM and 10 µM OA decreased the ROS levels; this could be related with the levels of CAT found in MCF7 cells in the present study (Figure 6b). MA did not alter the activity of CAT with respect to the control in MCF7 cells (Figure 6b).

Although there were no statistically significant differences in treated MDA-MB-231 cells, there was a slight decrease in the activity of CAT at 1 and 10 µM OA (Figure 6c).

### 2.7. Effects on H_2_O_2_-Induced DNA Damage 

To study the protective effect of these triterpenes against induced DNA injury, H_2_O_2_ was used to promote single-strand DNA breaks. The results are expressed as the percentage of Olive_TM for each cell line. Olive_TM incorporates a measure of both the smallest detectable size of migrating DNA (reflected in the comet tail length) and the number of relaxed/broken pieces (represented by the intensity of DNA in the tail), so this measure gives us information about the injury induced to DNA and the capacity for self-repair [29].

**Figure 7:**
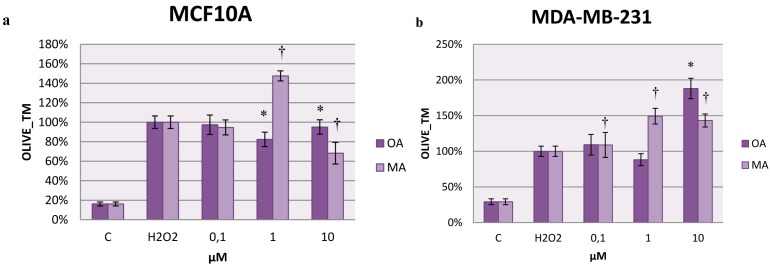
Olive_TM represented in MCF10A cells (**a**) and MDA-MB-231 cells (**b**) treated with OA or MA at 0.1 µM, 1 µM, and 10 µM for 4 h. Values represent the mean ± SEM of three independent experiments. Statistically significant differences are represented by (*) for OA and (†) for MA at *p* < 0.05 with respect to the H_2_O_2_ control.

Our results showed that for MCF10A cells, 1 µM OA protected against H_2_O_2_ injury to DNA, producing less DNA breaks than the control (Figure 7a). MA had the same effect at 10 µM, but it must be noted that at this concentration, MA was pro-apoptotic for the human mammary epithelial cells. Therefore, this result was likely due to cells that remained alive in the cytotoxicity and proliferation assay and were not affected by MA.

Although 1 µM MA did not have pro-apoptotic effects, it appeared to promote damage to DNA, which supports the results obtained for the detection of ROS levels after H_2_O_2_ addition. MA could act like a pro-oxidant in these cells, increasing the ROS levels in the first moments of treatment and resulting in damage to the DNA after addition of H_2_O_2_, consistent with our results. However, the effect of MA at this concentration did not remain the same over time, as the proliferation results have shown. 

In the MCF7 cells, MA did not show significant differences with respect to the control (data not shown). In the MDA-MB-231 cells, OA promoted an increase in Olive_TM at 10 µM, and although it was not statistically significant, this increase also occurred at 0.1 µM. MA induced more injury to the DNA, increasing the Olive_TM at all concentrations tested in MDA-MB-231 cells (Figure 7b). Consequently, for highly invasive breast cancer cells, with only 4 h of treatment, both compounds promoted a high extent of damage to the DNA. Therefore, the cytotoxic effects of oleanolic acid observed in MDA-MB-231 cells appeared to be connected with the increase observed in the ROS levels that in turn promoted damage to the DNA.

## 3. Discussion

OA and MA are two triterpenes present in several plants, including grapevines and olive trees and consequently in their fruits. It is well known that the Mediterranean diet plays a role in preventing breast cancer [8], and these foods are typically found in this diet. Several studies have suggested the antitumoral properties of AO and MA, but until now, there has not been scientific data about their chemopreventive activity in human breast cancer and in human mammary epithelial cells. The present study is focused on the effects of these two natural compounds on human breast cancer cells and on human mammary epithelial cells, which never were studied before.

The results obtained show that MA inhibited the growth of minimally invasive MCF7 human breast cancer cells only at the highest concentration tested. Thus, MA treatment does not alter the cell cycle or induce apoptosis at the concentrations used previously by our group [10] or in the present study. However, Janicke, *et al.* [30] indicated that MCF7 cells have lost caspase-3 due to a 47-base-pair deletion within exon 3 of the CASP-3 gene, and this deletion is required for DNA fragmentation and phosphatidylserine expression on the cell surface. Accordingly, in the present study, MCF7 cells did not experience apoptosis, as indicated by flow cytometry, nor did they have changes in DNA fragmentation by the comet assay, but a decrease in cell proliferation was observed with OA treatment [10] and MA treatment. Thus, MA, which in turn promoted a dramatic increase in the ROS levels inside MCF7 cells, may promote their death but through a pathway distinct from apoptosis. In fact, an increase in ROS levels could contribute to cell death in cancer cells [31].

Indeed, MA can promote apoptosis in HT29 colon cancer cells through ROS generation [32,33]. Therefore, the connection between the ROS levels and cell death appears to be established. Our results demonstrate that OA and MA promote DNA damage in MDA-MB-231 cells. Further in-depth studies focusing on the molecular mechanism of the effects of OA and MA in breast cancer cells must be performed to confirm this. It must be noted that for several assays, the MCF7 cells were treated with high-purity MA (purity >98%) because the present study shows differences in MCF7 cells not reported in the previous study [10], where the purity of MA was lower (>80%).

OA has been recently described to be pro-apoptotic in oestrogen receptor-negative/progesterone receptor-negative/HER2-negative (ER−/PR−/Her2−) breast cancer cells [34], and patients with an ER− genotype are considered to have more aggressive, highly invasive breast cancer than patients with an ER+ genotype [35]. Chu, *et al.* described the action of BN107 (an extract with several terpenoidal saponins similar to OA), which promotes apoptosis in MCF10A (ER−) and in MDA-MB-231 (ER−) cells [34]. They concluded that BN107 and OA are strong inhibitors of the Akt/mammalian target of rapamycin (mTOR) pathway, which could avoid chemoresistance development in ER− breast cancer cells. Our results show that although MCF10A cells are ER−, OA was not able to cause cell death at concentrations lower than 10 µM; at these concentrations, OA had antiproliferative effects in the highly invasive MDA-MB-231 human breast cancer cells. Based on these results, the effects of OA appear to not be related to ER expression; depending on the concentration used, OA is able to promote cell death in ER− cells (MDA-MB-231 and MCF10A) and ER+ cells (MCF7) [10].

OA has been shown to decrease the expression of Bcl-2 and increase the expression of Bax in B16F10 melanoma cells [36]. Perhaps OA exerts its effect in MDA-MB-231 cells by this pathway, which is related to oxidative mechanisms in the cell [27]. It is known that an increase in the ROS levels promotes apoptosis in breast cancer cells [37]. OA could increase the ROS levels in highly invasive cancer cells and could support the action of chemotherapies that increase oxidative stress inside cancer cells, which are usually used in more aggressive, highly invasive breast cancers.

Concentrations of OA and MA higher than 10 µM inhibited human mammary epithelial cell proliferation and promoted apoptosis over time, but lower concentrations even improved the proliferation of these human mammary epithelial cells. Hence, the concentration of the treatment used is an important consideration. Very few articles describe the bioavailability of these triterpenes in humans after intake [38,39,40]; but several studies confirm that OA can be absorbed (0.7% of total oral bioavailibity) by rats after intake, as well as MA which was observed even after 60 min of oral administration in rat’s plasma [27]. However, the concentration within the cells after the metabolism of these compounds is not described yet. Nevertheless, the concentration at which they are present in virgin olive oil is less than in other types of olive oils [5].

Our results showed that OA acts like an antioxidant in human mammary epithelial cells (MCF10A) *in vitro*. OA may decrease the oxidative stress of cells by enzymatic CAT activation. Furthermore, when oxidative stress was induced, the cells treated with OA had decreased levels of oxidative stress compared to the untreated cells. The irreversible injuries to DNA and proteins caused by oxidative stress are usually prevented by antioxidants [28]; along these lines, OA acts as an antioxidant for MCF10A cells, protecting the cells against oxidative DNA damage. Moreover, OA inhibited proliferation in MDA-MB-231 cells (highly invasive human breast cancer cells). 

For these reasons, we might consider that OA could have potential chemopreventive activity in human breast cancer: at low concentrations, OA is a natural compound that acts as an antioxidant and prevents oxidative DNA damage in human mammary epithelial cells. Additionally, it has antiproliferative effects in highly invasive cancer cells. This compound could be used as an adjuvant in breast cancer oxidative therapies, where it could maximize the effects of chemotherapy while protecting human mammary epithelial cells against the oxidative effects of cancer therapy. However, pharmacologic effects of OA have to be studied before assure this. 

Nevertheless, extreme caution should be applied in the extrapolation of the present *in vitro* results to potential clinical effects in humans. Further studies are needed to confirm both the chemopreventive capacity of OA and the differential mechanism of action on human mammary epithelial *vs* breast cancer cells suggested by the present study. 

## 4. Experimental Section

### 4.1. Chemicals

Oleanolic acid (OA) CAS [508-02-1] (purity ≥97%) was purchased from Extrasynthese (Genay, France). Maslinic acid (MA) CAS [4373-41-5] (purity ≥98%) was obtained from Cayman Chemical (Ann Arbor, MI, USA). The following were purchased from Sigma-Aldrich Co. (St Louis, MO, USA): Hepes solution; sodium pyruvate solution; 100× non-essential amino acid mixture (NEAA); 2,7-dichlorofluorescin diacetate (DCFH-DA) CAS [4091-99-0] (purity ≥97%); dimethyl sulfoxide (DMSO); 2,3-Bis(2-methoxy-4-nitro-5-sulfophenyl)-2*H*-tetrazolium-5-carboxanilide inner salt (XTT sodium salt) (purity ≥90%); *N*-Methylphenazonium methyl sulfate (PMS) (purity ≥98%); phosphate buffered saline (PBS); (*S*)-(+)-camptothecin (CPT) CAS [7689-03-4] (purity ≥90%); and Triton X-100. Foetal Bovine Serum (FBS) was obtained from PAA Laboratories GmbH (Pasching, Austria). TrypLE Express, HuMEC ready medium, minimum essential medium with Eagle’s salts (MEM) and Phenol-Red-free Roswell Park Memorial Institute 1640 medium (RPMI) were obtained from Gibco^®^ Life Technologies Ltd (Paisley, UK). Dry methanol (max 0.005%) and absolute ethanol PRS were purchased from Panreac Quimica S.L.U. (Barcelona, Spain). The CellTiter-Blue^®^ Cell Viability Assay was obtained from Promega Corporation (Madison, WI, USA). Phosphate buffered saline (1X, Dulbecco’s) (PBS) was purchased from Applichem GmbH (Gatersleben, Germany). Culture plates were obtained from Starlab (Hamburg, Germany). The PI/RNase staining buffer kit was obtained from BD Biosciences Pharmingen (San Diego, CA, USA). The Annexin-V FITC kit was purchased from Miltenyi Biotec (Cologne, Germany). The comet assay kit was obtained from Trevigen, Inc. (Helgerman CT, Gaithersburg, MD, USA). The catalase assay kit was purchased by Merck KGAA (Darmstadt, Germany). 

### 4.2. Cell Culture and Treatments

Highly invasive MDA-MB-231 (ATCC^®^ Number: HTB-26™) human breast cancer cells (oestrogen and progesterone receptor-negative), minimally invasive MCF7 (ATCC^®^ Number: HTB-22™) human breast cancer cells (oestrogen and progesterone receptor-positive), and immortalized MCF10A (ATCC^®^ Number: CRL-10317™) human mammary epithelial cells (oestrogen receptor-negative), were obtained from American Type Culture Collection (ATCC, Manassas, VA, USA). Breast cancer cells (MCF7 and MDA-MB-231) were grown as monolayer cultures in MEM supplemented with 10% FBS, 1% Hepes buffer, 1% sodium pyruvate and 1% NEAA. Human mammary epithelial cells (MCF10A) were grown in HuMEC Ready Medium. Cell lines were maintained at 37 °C in a humidified atmosphere with 5% CO_2_. The cells were routinely subcultured using TrypLE Express solution. Cells in the exponential growth phase were used for all experiments. Except for the assays which specify differently, the cells were treated with 0.1 µM, 1 µM, or 10 µM oleanolic acid (OA) and maslinic acid (MA) for 4 h.

### 4.3. Cytotoxicity Assay

Cell survival, measured as the cellular growth of the treated cells *vs.* the untreated controls, was carried out in MCF10A, MCF7 and MDA-MB-231 cells using an XTT-based assay according to Scudiero, *et al.* [41], with some modifications. Briefly, cells were seeded into 96-well culture plates in a total volume of 100 µL per well (5 × 10^3^ cells/well for MDA-MB-231 and MCF7 cells and 2.5 × 10^3^ cells/well for MCF10A cells). After an overnight incubation to allow for cell attachment, 100 µL of fresh medium was added containing increasing concentrations from 0.001 µM to 100 µM OA or MA. After 24 h, the cells were incubated with XTT in Phenol-Red-free RPMI medium for 3 h at 37 °C with 5% CO_2_, and the absorbance was measured at a 450 nm wavelength (620 nm as a reference) in a plate reader (TECAN GENios Plus). The cell viability was calculated using the formula:

% viable cells = [A(treated cells)/A(control)] × 100
(1)

where A is the difference in absorbance between optical density units (A = OD_450_ − OD_620_). All measurements were performed in quadruplicate, and each experiment was repeated at least three times. As a vehicle control, the cells were treated with EtOH at the highest concentration of OA and MA used.

### 4.4. Cell Proliferation Assay

Cell proliferation, measured as the cellular growth of the treated cells *vs.* the untreated controls, was carried out using a CellTiter-Blue Cell Viability Assay. Briefly, the cells were seeded into 96-well culture plates at 2 × 10^3^ cells/well for MCF7 cells, 1 × 10^3^ cells/well for MDA-MB-231 cells and 0.5 × 10^3^ cells/well for MCF10A cells. After an overnight incubation to allow for cell attachment, the medium was removed and replaced with fresh medium containing OA or MA from 0.01 µM to 100 µM. The plates were incubated for 24, 48 or 72 h, followed by a 72 h, 48 h and 24 h proliferation period (incubation with fresh medium without OA or MA), respectively. At these three time points, the plates were incubated with CellTiter-Blue Cell Viability for 3 h at 37 °C with 5% CO_2_ and the relative fluorescence units were measured in a plate reader (TECAN GENios Plus) (Ex. λ_485_/Em. λ_595_, Gain 60). The cell viability was calculated using the formula:

% viable cells = [A(treated cells)/A(control)] × 100 (2)

where A are the relative fluorescence units for each sample. All measurements were performed in triplicate, and each experiment was repeated at least three times. As a vehicle control, the cells were treated with EtOH at the highest concentration of OA or MA used.

### 4.5. Cell Cycle Assay

The cells were seeded in 12-well culture plates (1 × 10^5^ cells/well for MDA-MB-231 and MCF7 cells and 0.5 × 10^5^ cells/well for MCF10A cells) and incubated overnight to allow for cell attachment. Next, the cells were treated with 0.1 µM, 1 µM, or 10 µM OA or MA for 24 h; the cells were harvested with TrypLE Express and washed with 1× PBS (Ca^2+^/Mg^2+^ free) (300× *g*, 10 min at 4 °C). Finally, the cells were fixed with cold 70% ethanol and stored at −20 °C for at least 24 h. Subsequent to propidium iodide labelling (PI/RNase Staining Buffer), the cells were analysed by flow cytometry (EPICS XL-MCL, Beckman Coulter, Spain). The FlowJo program (v5.7.2, FlowJo LLC data analysis software, Ashland, OR, USA) was used to calculate the percentage of cells in the G0/G1, S and G2/M phases. Each experiment was independently repeated at least three times. 

### 4.6. Apoptosis Assay

The percentage of apoptotic cells was determined using a double staining assay with FITC-conjugated Annexin V and propidium iodide (PI). Briefly; the cells were seeded in 12-well culture plates (1 × 10^5^ cells/well for MDA-MB-231 and MCF7 cells and 0.5 × 10^5^ cells/well for MCF10A cells) and incubated overnight to allow for cell attachment. After cell exposure to OA or MA at 0.1 µM, 1 µM, or 10 µM for 24 h; the cells were harvested with TrypLE Express; washed twice in cold 1× PBS (Ca^2+^/Mg^2+^ free) (300× *g*; 10 min at 4 °C) and resuspended in 100 µL of Annexin Binding Buffer. The cells were stained with 5 µL Annexin V-FITC and 2 µL PI solution; gently vortexed and incubated for 15 min at room temperature in the dark before flow cytometric analysis. As a positive control; the cells were treated with 1 µM camptothecin (CPT). Each experiment was independently repeated at least three times.

### 4.7. Detection of Intracellular Reactive Oxygen Species

Intracellular reactive oxygen species (ROS) levels were measured after OA or MA treatment using the cell-permeable fluorescent probe 2,7-dichlorofluorescin diacetate (DCFH-DA), as previously described by Warleta, *et al.* [11], with some modifications. Briefly, the cells were seeded on 96-well plates (5 × 10^3^ cells/well for MDA-MB-231 and MCF7 cells and 2.5 × 10^3^ cells/well for MCF10A cells), and after incubation with the treatments, DCFH-DA (100 µM) was added for 30 min at 37 °C with 5% CO_2_. The fluorescence was read in a plate reader for 30 min (Ex. λ485/Em. λ535, Gain 60). The intracellular ROS level percentage was calculated as follows:

F = [(F(t = 30) – F(t = 0))/F(t = 0) × 100]
(3)

where F(t = 0) is the fluorescence at t = 0 min and F(t = 30) the fluorescence at t = 30 min. It has been described that the addition of H_2_O_2_ increases oxidative stress in cultured cells and directly damages DNA [42]. To evaluate the protective capacity of OA and MA against induced oxidative stress, 500 µM H_2_O_2_ was added 30 min before the fluorescence quantification.

All tests were run in triplicate for each experimental condition, and each experiment was repeated at least three times. All experiments were conducted using iron-free media (MEM and HuMEC). 

### 4.8. Determination of Catalase (CAT) Activity 

The cells were seeded into a 6-well plate at 0.5 × 10^6^ cells/mL for MCF10A, MDA-MB-231 and MCF7 cells. The cells were incubated overnight for cell attachment. Then, the medium was changed to fresh medium containing OA or MA. The assay was performed according to the manufacturer’s protocol for the determination of catalase enzymatic activity. 

### 4.9. Alkaline Single-Cell Gel Electrophoresis (Comet Assay)

The cells were seeded into 12-well plates (1 × 10^5^ cells/well for MDA-MB-231 cells and MCF7 cells and 0.5 × 10^5^ cells/well for MCF10A cells) and incubated overnight for cell attachment. Then, the cells were treated with OA and MA. Finally, the cells were scraped and washed twice (300× *g*, 10 min, 4 °C) with cold 1× PBS (Ca^2+^/Mg^2+^ free) and suspended in 1 mL of cold 1× PBS. To evaluate the ability of OA and MA to protect against oxidative DNA damage, the cells were exposed for 10 min to 50 µM H_2_O_2_ at 4 °C. After that, the comet assay was performed according to Warleta, *et al.* [11].

### 4.10. Slide Scoring and Analysis

DNA strand breaks were examined using a fluorescence microscope (Zeiss Axiovert 200) equipped with a Luca EMCCD camera (Andor Technology, Belfast, UK) under 494 nm excitation and 521 nm emission wavelengths using the Komet 5.5 software package (Kinetic Imaging Ltd., Liverpool, UK). Twenty-five cell images were randomly characterized per sample using 20× magnification. The relative fluorescence between the head and tail through the olive tail moment (Olive_TM) was used to determine DNA damage. Olive_TM is defined as the product of the Tail Moment Length and the fraction of DNA in the tail:

Olive_TM = [(tail (mean) − head (mean)) × tail (% DNA)]/100
(4)

### 4.11. Statistical Analysis

The results are displayed as the mean of at least three independent experiments (± SEM), and the results are expressed as a percentage relative to the untreated control, which was set as 100%. Statistical analysis was performed using a one-way analysis of variance (ANOVA) followed by Fisher’s LSD test. Values of *p* < 0.05 were considered significant. STATGRAPHICS Plus 5.1 statistical software (Statpoint Technologies, Inc., Warrenton, VA, USA) was used for the statistical analysis.

## References

[1] Zhang Y., Jayaprakasam B., Seeram N.P., Olson L.K., DeWitt D., Nair M.G. (2004). Insulin Secretion and Cyclooxygenase Enzyme Inhibition by Cabernet Sauvignon Grape Skin Compounds. J. Agric. Food Chem..

[2] Glensk M., Glinski J.A., Wlodarczyk M., Stefanowicz P. (2014). Determination of Ursolic and Oleanolic Acid in Sambuci Fructus. Chem. Biodivers..

[3] Szakiel A., Mroczek A. (2007). Distribution of Triterpene Acids and their Derivatives in Organs of Cowberry (*Vaccinium vitis-idaea* L.) Plant. Acta Biochim. Pol..

[4] Szakiel A., Paczkowski C., Koivuniemi H., Huttunen S. (2012). Comparison of the Triterpenoid Content of Berries and Leaves of Lingonberry *Vaccinium vitis-idaea* from Finland and Poland. J. Agric. Food Chem..

[5] Allouche Y., Jimenez A., Uceda M., Aguilera M.P., Gaforio J.J., Beltran G. (2009). Triterpenic Content and Chemometric Analysis of Virgin Olive Oils from Forty Olive Cultivars. J. Agric. Food Chem..

[6] Pensec F., Paczkowski C., Grabarczyk M., Wozniak A., Benard-Gellon M., Bertsch C., Chong J., Szakiel A. (2014). Changes in the Triterpenoid Content of Cuticular Waxes during Fruit Ripening of Eight Grape (*Vitis vinifera*) Cultivars Grown in the Upper Rhine Valley. J. Agric. Food Chem..

[7] Yunoki K., Sasaki G., Tokuji Y., Kinoshita M., Naito A., Aida K., Ohnishi M. (2008). Effect of Dietary Wine Pomace Extract and Oleanolic Acid on Plasma Lipids in Rats Fed High-Fat Diet and its DNA Microarray Analysis. J. Agric. Food Chem..

[8] Buckland G., Travier N., Cottet V., Gonzalez C.A., Lujan-Barroso L., Agudo A., Trichopoulou A., Lagiou P., Trichopoulos D., Peeters P.H. (2013). Adherence to the Mediterranean Diet and Risk of Breast Cancer in the European Prospective Investigation into Cancer and Nutrition Cohort Study. Int. J. Cancer.

[9] Patlolla J.M., Rao C.V. (2012). Triterpenoids for Cancer Prevention and Treatment: Current Status and Future Prospects. Curr. Pharm. Biotechnol..

[10] Allouche Y., Warleta F., Campos M., Sanchez-Quesada C., Uceda M., Beltran G., Gaforio J.J. (2011). Antioxidant, Antiproliferative, and Pro-Apoptotic Capacities of Pentacyclic Triterpenes found in the Skin of Olives on MCF-7 Human Breast Cancer Cells and their Effects on DNA Damage. J. Agric. Food Chem..

[11] Warleta F., Campos M., Allouche Y., Sanchez-Quesada C., Ruiz-Mora J., Beltran G., Gaforio J.J. (2010). Squalene Protects against Oxidative DNA Damage in MCF10A Human Mammary Epithelial Cells but Not in MCF7 and MDA-MB-231 Human Breast Cancer Cells. Food Chem. Toxicol..

[12] Warleta F., Quesada C.S., Campos M., Allouche Y., Beltran G., Gaforio J.J. (2011). Hydroxytyrosol Protects Against Oxidative DNA Damage in Human Breast Cells. Nutrients.

[13] Sanchez-Quesada C., Lopez-Biedma A., Gaforio J.J. (2015). The Differential Localization of a Methyl Group Confers a Different Anti-Breast Cancer Activity to Two Triterpenes Present in Olives. Food Funct..

[14] Allouche Y., Beltran G., Gaforio J.J., Uceda M., Mesa M.D. (2010). Antioxidant and Antiatherogenic Activities of Pentacyclic Triterpenic Diols and Acids. Food Chem. Toxicol..

[15] Senthil S., Sridevi M., Pugalendi K.V. (2007). Cardioprotective Effect of Oleanolic Acid on Isoproterenol-Induced Myocardial Ischemia in Rats. Toxicol. Pathol..

[16] Graham V.S., Lawson C., Wheeler-Jones C.P., Perona J.S., Ruiz-Gutierrez V., Botham K.M. (2012). Triacylglycerol-Rich Lipoproteins Derived from Healthy Donors Fed Different Olive Oils Modulate Cytokine Secretion and Cyclooxygenase-2 Expression in Macrophages: The Potential Role of Oleanolic Acid. Eur. J. Nutr..

[17] Li C., Yang Z., Zhai C., Qiu W., Li D., Yi Z., Wang L., Tang J., Qian M., Luo J. (2010). Maslinic Acid Potentiates the Anti-Tumor Activity of Tumor Necrosis Factor Alpha by Inhibiting NF-kappaB Signaling Pathway. Mol. Cancer..

[18] Park S.Y., Nho C.W., Kwon D.Y., Kang Y.H., Lee K.W., Park J.H. (2013). Maslinic Acid Inhibits the Metastatic Capacity of DU145 Human Prostate Cancer Cells: Possible Mediation via Hypoxia-Inducible Factor-1alpha Signalling. Br. J. Nutr..

[19] Wang X., Bai H., Zhang X., Liu J., Cao P., Liao N., Zhang W., Wang Z., Hai C. (2013). Inhibitory Effect of Oleanolic Acid on Hepatocellular Carcinoma via ERK-p53-Mediated Cell Cycle Arrest and Mitochondrial-Dependent Apoptosis. Carcinogenesis.

[20] Wei J., Liu M., Liu H., Wang H., Wang F., Zhang Y., Han L., Lin X. (2013). Oleanolic Acid Arrests Cell Cycle and Induces Apoptosis via ROS-Mediated Mitochondrial Depolarization and Lysosomal Membrane Permeabilization in Human Pancreatic Cancer Cells. J. Appl. Toxicol..

[21] Rufino-Palomares E.E., Reyes-Zurita F.J., Garcia-Salguero L., Mokhtari K., Medina P.P., Lupianez J.A., Peragon J. (2013). Maslinic Acid, a Triterpenic Anti-Tumoural Agent, Interferes with Cytoskeleton Protein Expression in HT29 Human Colon-Cancer Cells. J. Proteomics.

[22] Sanchez-Tena S., Reyes-Zurita F.J., Diaz-Moralli S., Vinardell M.P., Reed M., Garcia-Garcia F., Dopazo J., Lupianez J.A., Gunther U., Cascante M. (2013). Maslinic Acid-Enriched Diet Decreases Intestinal Tumorigenesis in Apc(Min/+) Mice through Transcriptomic and Metabolomic Reprogramming. PLoS ONE.

[23] Shan J.Z., Xuan Y.Y., Ruan S.Q., Sun M. (2011). Proliferation-Inhibiting and Apoptosis-Inducing Effects of Ursolic Acid and Oleanolic Acid on Multi-Drug Resistance Cancer Cells *in Vitro*. Chin. J. Integr. Med..

[24] He X., Wang Y., Hu H., Zhang Z. (2012). *In Vitro* and *in Vivo* Antimammary Tumor Activities and Mechanisms of the Apple Total Triterpenoids. J. Agric. Food Chem..

[25] Chakravarti B., Maurya R., Siddiqui J.A., Bid H.K., Rajendran S.M., Yadav P.P., Konwar R. (2012). *In Vitro* Anti-Breast Cancer Activity of Ethanolic Extract of Wrightia Tomentosa: Role of Pro-Apoptotic Effects of Oleanolic Acid and Urosolic Acid. J. Ethnopharmacol..

[26] Ponou B.K., Teponno R.B., Ricciutelli M., Nguelefack T.B., Quassinti L., Bramucci M., Lupidi G., Barboni L., Tapondjou L.A. (2011). Novel 3-Oxo- and 3,24-Dinor-2,4-Secooleanane-Type Triterpenes from Terminalia Ivorensis A. Chem. Biodivers..

[27] Sanchez-Quesada C., Lopez-Biedma A., Warleta F., Campos M., Beltran G., Gaforio J.J. (2013). Bioactive Properties of the Main Triterpenes found in Olives, Virgin Olive Oil, and Leaves of *Olea europaea*. J. Agric. Food Chem..

[28] Saeidnia S., Abdollahi M. (2013). Antioxidants: Friends or Foe in Prevention or Treatment of Cancer: The Debate of the Century. Toxicol. Appl. Pharmacol..

[29] Singh N.P., McCoy M.T., Tice R.R., Schneider E.L. (1988). A Simple Technique for Quantitation of Low Levels of DNA Damage in Individual Cells. Exp. Cell Res..

[30] Janicke R.U., Sprengart M.L., Wati M.R., Porter A.G. (1998). Caspase-3 is required for DNA Fragmentation and Morphological Changes Associated with Apoptosis. J. Biol. Chem..

[31] Bonnet S., Archer S.L., Allalunis-Turner J., Haromy A., Beaulieu C., Thompson R., Lee C.T., Lopaschuk G.D., Puttagunta L., Bonnet S. (2007). A Mitochondria-K^+^ Channel Axis is Suppressed in Cancer and its Normalization Promotes Apoptosis and Inhibits Cancer Growth. Cancer Cell..

[32] Reyes-Zurita F.J., Rufino-Palomares E.E., Lupianez J.A., Cascante M. (2009). Maslinic Acid, a Natural Triterpene from *Olea europaea* L., Induces Apoptosis in HT29 Human Colon-Cancer Cells via the Mitochondrial Apoptotic Pathway. Cancer Lett..

[33] Reyes-Zurita F.J., Pachon-Pena G., Lizarraga D., Rufino-Palomares E.E., Cascante M., Lupianez J.A. (2011). The Natural Triterpene Maslinic Acid Induces Apoptosis in HT29 Colon Cancer Cells by a JNK-p53-Dependent Mechanism. BMC Cancer.

[34] Chu R., Zhao X., Griffin C., Staub R.E., Shoemaker M., Climent J., Leitman D., Cohen I., Shtivelman E., Fong S. (2010). Selective Concomitant Inhibition of mTORC1 and mTORC2 Activity in Estrogen Receptor Negative Breast Cancer Cells by BN107 and Oleanolic Acid. Int. J. Cancer.

[35] Sheikh M.S., Garcia M., Pujol P., Fontana J.A., Rochefort H. (1994). Why are Estrogen-Receptor-Negative Breast Cancers More Aggressive than the Estrogen-Receptor-Positive Breast Cancers?. Invasion Metastasis.

[36] Pratheeshkumar P., Kuttan G. (2011). Oleanolic Acid Induces Apoptosis by Modulating p53, Bax, Bcl-2 and Caspase-3 Gene Expression and Regulates the Activation of Transcription Factors and Cytokine Profile in B16F. J. Environ. Pathol. Toxicol. Oncol..

[37] Vera-Ramirez L., Sanchez-Rovira P., Ramirez-Tortosa M.C., Ramirez-Tortosa C.L., Granados-Principal S., Lorente J.A., Quiles J.L. (2011). Free Radicals in Breast Carcinogenesis, Breast Cancer Progression and Cancer Stem Cells. Biological Bases to Develop Oxidative-Based Therapies. Crit. Rev. Oncol. Hematol..

[38] Song M., Hang T.J., Wang Y., Jiang L., Wu X.L., Zhang Z., Shen J., Zhang Y. (2006). Determination of Oleanolic Acid in Human Plasma and Study of its Pharmacokinetics in Chinese Healthy Male Volunteers by HPLC Tandem Mass Spectrometry. J. Pharm. Biomed. Anal..

[39] Rada M., Ruiz-Gutierrez V., Guinda A. (2011). Determination of Triterpenic Acids in Human Serum by High-Performance Liquid Chromatography: Triterpenoid Interaction with Serum Protein. J. Agric. Food Chem..

[40] Lozano-Mena G., Juan M.E., Garcia-Granados A., Planas J.M. (2012). Determination of Maslinic Acid, a Pentacyclic Triterpene from Olives, in Rat Plasma by High-Performance Liquid Chromatography. J. Agric. Food Chem..

[41] Scudiero D.A., Shoemaker R.H., Paull K.D., Monks A., Tierney S., Nofziger T.H., Currens M.J., Seniff D., Boyd M.R. (1988). Evaluation of a Soluble tetrazolium/formazan Assay for Cell Growth and Drug Sensitivity in Culture using Human and Other Tumor Cell Lines. Cancer Res..

[42] Lee D.H., Lim B.S., Lee Y.K., Yang H.C. (2006). Effects of Hydrogen Peroxide (H_2_O_2_) on Alkaline Phosphatase Activity and Matrix Mineralization of Odontoblast and Osteoblast Cell Lines. Cell Biol. Toxicol..

